# Antifibrotic Effects of *Thymus syriacus* Essential Oil in Bleomycin-Induced Pulmonary Fibrosis via Suppression of the TGF-β1/Smad2 Axis

**DOI:** 10.3390/ijms27031401

**Published:** 2026-01-30

**Authors:** Pınar Aksoy, Önder Yumrutaş, Muhittin Doğan, Pınar Yumrutaş, Mehmet Sökücü, Mustafa Pehlivan

**Affiliations:** 1 Department of Biology, Faculty of Science, Gaziantep University, 27010 Gaziantep, Turkey; 2 Department of Medical Biology, Faculty of Medicine, Adıyaman University, 02100 Adiyaman, Turkey; 3 Department of Medical Pharmacology, Faculty of Medicine, Adıyaman University, 02100 Adiyaman, Turkey; 4 Department of Pathology, Faculty of Medicine, Sanko University, 27090 Gaziantep, Turkey

**Keywords:** antifibrotic effect, pulmonary fibrosis, TGF-β1/SMAD2, *Thymus syriacus*, Thymol

## Abstract

Background: Pulmonary fibrosis (PF) is an irreversible interstitial lung disease in which the TGF-β/SMAD signaling pathway plays a critical role in its pathogenesis. Due to the anti-inflammatory and antioxidant properties of *Thymus* species, it is hypothesized that they may suppress pulmonary fibrosis by modulating the TGF-β/SMAD pathway. This study aimed to investigate the potential antifibrotic effects of *Thymus syriacus* essential oil (TS) on the TGF-β/SMAD pathway in bleomycin-induced PF. Methods: PF was induced with bleomycin, and TS was administered at concentrations of 50 and 100 mg/mL for 28 days. mRNA and protein levels of TGF-β1, SMAD2, COL1, and α-SMA in lung tissues isolated were analyzed using real-time PCR and ELISA. TNF-α levels in BALF were measured by ELISA. ROS and MDA levels in lung issues were determined using 2,7-DHCFDA and TBARS tests, respectively. Histopathological evaluation was performed using Hematoxylin–Eosin and Masson’s trichrome staining. Blood samples were analyzed for kidney, liver, and cardiac toxicity markers. The chemical composition of TS was determined by GC–MS. Results: TS significantly reduced levels of TGF-β1, SMAD2, COL1, α-SMA, TNF-α, ROS and MDA compared to the BLM group. PF alterations were markedly attenuated by TS treatment. Thymol, p-cymene and carvacrol were identified as major constituents of TS. Conclusion: Overall, TS alleviates pulmonary fibrosis by suppressing the TGF-β/SMAD2 signaling pathway.

## 1. Introduction

Pulmonary fibrosis is a fatal form of interstitial lung disease and is chronic, progressive, and irreversible [1]. It is characterized by the progressive accumulation of fibroblasts and extracellular matrix in the lung [2]. The fibrotic process is intricately linked to chronic inflammation and metabolic homeostasis, and the equilibrium between oxidant and antioxidant systems serves as a pivotal modulator in regulating these pathways [3]. This suggests that the interaction between aberrant fibroblast activation and inflammatory and epithelial signaling pathways plays a critical role in the progression of the disease [4].

Although smoking is known to be a triggering factor in the development of fibrosis [5], genetic and environmental factors may also lead to the formation of pulmonary fibrosis [6]. In addition, pulmonary fibrosis can be triggered by viral infections, most notably herpesviruses. Recent studies have highlighted that SARS-CoV-2 (COVID-19), Torque teno virus and Epstein–Barr virus have the potential to induce fibrotic changes in the lungs [7,8]. Additionally, several comorbidities including lung cancer, atherosclerosis, gastroesophageal reflux, and diastolic dysfunction have been implicated in the pathogenesis of pulmonary fibrosis. The TGF-β/SMAD (Transforming growth factor-beta/Suppressor of Mothers Against Decapentaplegic 2) axis is established as one of the most fundamental signaling pathways driving the development of fibrosis. The SMAD family signaling cascade plays a pivotal role in fibrogenesis, serving as an essential driver for the activation of the myofibroblast phenotype, stimulation of extracellular matrix synthesis, and the modulation of integrin expression [9]. Specifically, TGF-β-mediated phosphorylation of Smad2 and Smad3 triggers their translocation into the nucleus, where they orchestrate the transcription of fibrosis-related target genes such as MMP1, collagen I, and α-SMA [10].

Pirfenidone, a standard therapeutic agent for pulmonary fibrosis, has been shown to attenuate fibroblast proliferation and to suppress the mRNA and protein expression of TGF-β-induced α-smooth muscle actin (α-SMA) and procollagen I (COL1) [11]. Furthermore, evidence suggests that pirfenidone inhibits TGF-β signaling, thereby diminishing collagen deposition and further hindering the expansion of the fibroblast population [12].

Despite to its antifibrotic efficacy, pirfenidone is associated with a range of adverse events including gastrointestinal, hepatic, and skin-related adverse effects. Moreover, its clinical utility is often complicated by significant drug–drug interactions [13]. Although pirfenidone is a mainstay in the clinical management of pulmonary fibrosis, it remains insufficient for the treatment or suppression of this currently incurable disease, thereby underscoring the urgent need for novel antifibrotic agents by minimal adverse effects.

Plants serve as a vast reservoir of secondary metabolites that typically offer lower systemic toxicity than synthetic alternatives. Due to their inherent antioxidant and anti-inflammatory properties, these natural compounds are increasingly recognized as viable candidates for suppressing the pathological cascades of pulmonary fibrosis induced by oxidative stress and chronic inflammation. Previous studies have demonstrated that various plant-derived compounds contribute to the suppression of fibrosis by modulating key signaling molecules, including TGF-β1, SMAD2, SMAD3, α-SMA, COL1 and TNF-α. These factors plays a critical role in epithelial–mesenchymal transition (EMT) and extracellular matrix aggregation (ECM) during fibrogenesis [14]. Furthermore, evidence from a study on human airway granulation fibroblasts indicated that the TGF-β/SMAD2 signaling pathway can be inhibited by phytochemical compounds [15].

The genus *Thymus*, belonging to the Lamiaceae, comprises approximately 214 species and is primarily distributed across North Africa, Europe, and temperate Asia regions [16]. Compounds such as thymol obtained from *Thymus* species are used in the pharmaceutical, food, and cosmetic industries due to their antimicrobial, antioxidant, anticarcinogenic, anti-inflammatory, and antispasmodic activities [17]. Although previous studies have reported antifibrotic roles of *Thymus* species in liver and lung fibrosis, these studies are still in their infancy and remain limited in scope [18,19]. While the essential oils of *Thymus* species contain a variety of chemical compounds, no studies have yet investigated their antifibrotic effects specifically on pulmonary fibrosis. In light of these findings, it is hypothesized that the phytochemical constituents of TS act synergistically to mitigate pulmonary fibrosis via the downregulation of the TGF-β/SMAD signaling pathway. Within this context, the present study aims to elucidate the effects of TS, rich in bioactive terpenoid compounds, on the TGF-β/SMAD2 signaling pathway, while also evaluating its regulatory role in the expression of α-SMA, collagen I, and the proinflammatory cytokine TNF-α.

## 2. Results

### 2.1. Assessment of Body Weight and Lung Index Parameters

**Figure 1:**
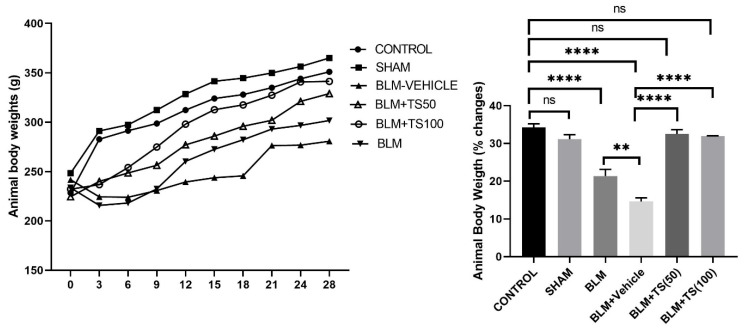
Body weights of animals measured over 28 days and percentage changes in body weight. The effects of BLM and TS administrations on animal body weights were determined by weighing the animals every three days from day 0 until the end of the experiment. “**”: *p* < 0.01, “****”: *p* =< 0.0001 and ns: not significant.

On the day of BLM administration, animals were weighed, and starting three days after BLM treatment, saline, vehicle, TS-50, and TS-100 were administered. Body weights were recorded every three days until the end of the experiment. The body weight changes and total body weight percentages for all groups are presented in Figure 1. As shown in the Figure 1, control (34.29%) and sham (31.14%) groups gained weight throughout the experimental period, with no significant difference observed between two groups in terms of percentage body weight change (*p* = 0.1839). In contrast, the BLM (21.36%) and BLM + Vehicle (14.6%) groups exhibited a significantly lower percentage of weight gain (*p* < 0.0001 for both groups). However, treatment with the BLM + TS50 (32.50%) and BLM + 100 (31.93%) significantly attenuated this effect, resulting in greater body weight increases compared to the BLM and BLM + Vehicle groups. Furthermore, the percentage of body weight gain in the BLM + TS50 (32.50%) and BLM + TS100 (31.93%) groups was similar to that of the control group (*p* = 0.6347 and *p* = 0.3966, respectively).

**Figure 2:**
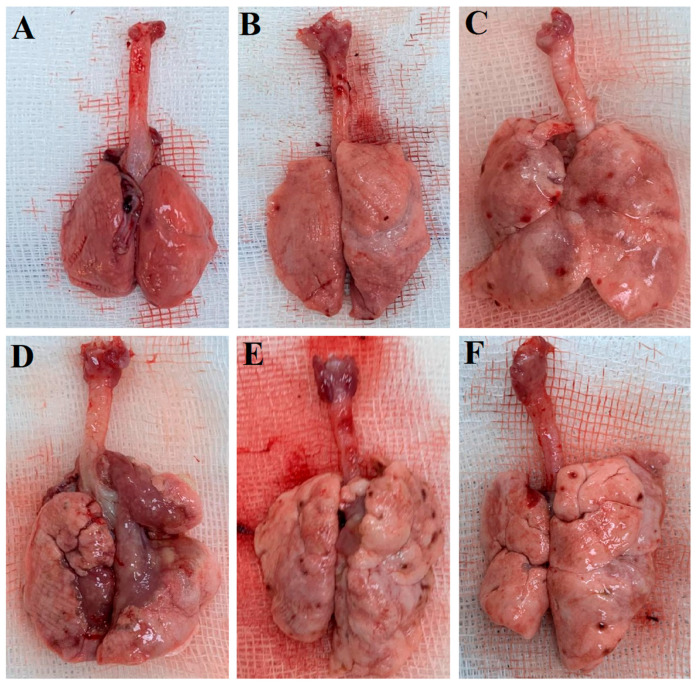
The lung images of the control (**A**), sham (**B**), BLM (**C**), BLM + Vehicle (**D**), BLM + TS50 (**E**), and BLM + TS100 (**F**) groups.

Figure 2 displays macroscopic lung images of the experimental groups obtained after sacrifice on day 28. As shown in Figure 2, the control and sham groups exhibit a normal physiological appearance with no visible pathological changes. In contrast, fibrotic areas are clearly apparent in the BLM + TS50 and BLM + TS100 groups. While fibrotic lesions are also present in the BLM + TS50 and BLM + TS100 groups, these areas are notably less extensive and less severe compared to those in the BLM and BLM + Vehicle groups.

**Figure 3:**
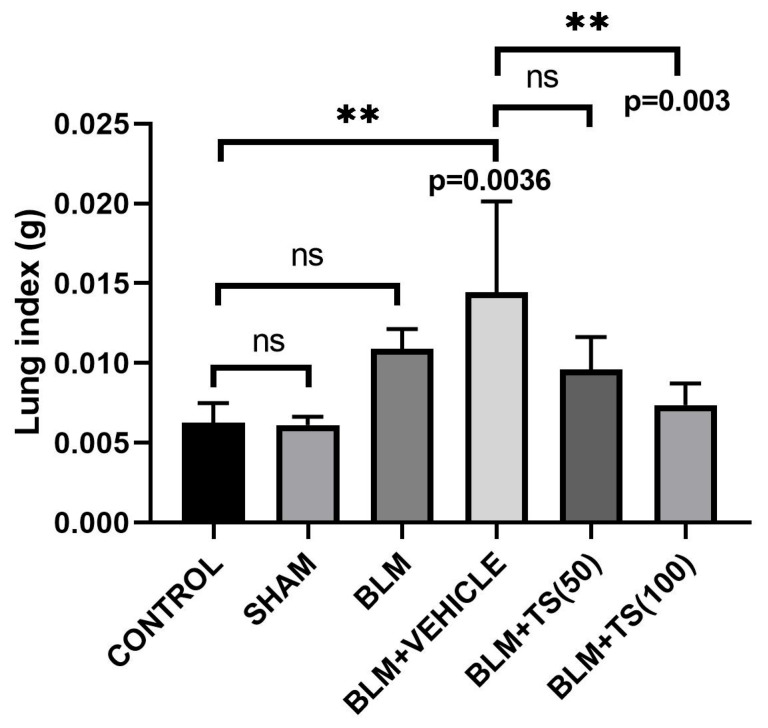
Lung index scores. Lung index serve as an indicator of the severity of bleomycin-induced injury and illustrates the efficacy of the therapeutic intervention. ns: no statistically significant difference (*p* > 0.05).

Furthermore, lung index, calculated as the ratio of lung weight to the final body weight, is presented in Figure 3. The results indicate that the lung index in the BLM and BLM + Vehicle groups was higher than those observed in the control and TS-treated groups. Compared with the control group, the increase in the lung index in the BLM group did not statistically significance (*p* = 0.1286), whereas the increase in the BLM + Vehicle group was statistically significant (*p* = 0.0036). Notably, a significant reduction in the lung index was observed in the BLM + TS100 group (*p* = 0.003). In contrast, while the BLM + TS50 group showed a decrease in the lung index, the change was not statistically significant (*p* = 0.0814).

### 2.2. Real-Time PCR Analysis of TGF-β1, SMAD2, COL1, and α-SMA mRNA Levels in Lung Tissues

**Figure 4:**
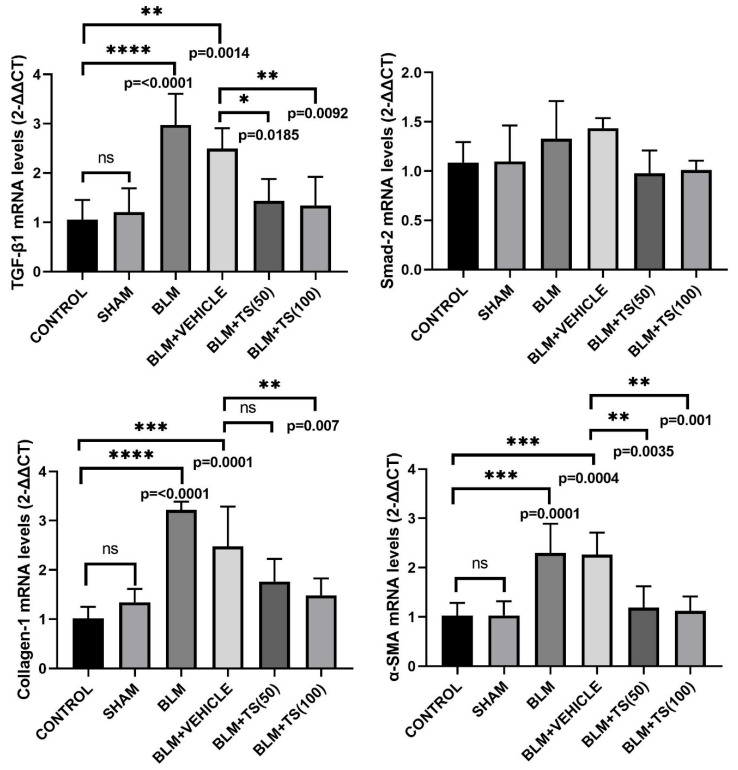
TGF-β1, SMAD2, COL1, and α-SMA mRNA levels in lung tissues. BLM administration significantly upregulated TGF-β1, COL1, and α-SMA mRNA levels in lung tissues compared to the control and sham groups. TS treatment at doses of 50 and 100 mg/kg markedly downregulated the mRNA levels of these fibrosis-associated genes. Data are expressed as relative mRNA levels calculated using the 2^−ΔΔCT^ method. ns: no statistically significant difference (*p* > 0.05).

The mRNA levels of TGF-β1, SMAD2, COL1, and α-SMA in lung tissues were determined using the Real-Time PCR method, with the results presented in Figure 4. As shown in Figure 4, TGF-β1 mRNA level remained unchanged in the control and sham groups. However, TGF-β1 mRNA levels were significantly upregulated in the BLM (2.97-fold) and BLM + Vehicle (2.48-fold) groups compared to the control group (*p* < 0.0001 and *p* = 0.0014, respectively). Notably, compared to the BLM + Vehicle group, TGF-β1 level was significantly downregulated in the BLM + TS50 (0.57-fold) and BLM + TS100 (0.54-fold) groups (*p* = 0.0185 and *p* = 0.0092, respectively). Regarding SMAD2 mRNA levels, an upregulation was observed in the BLM and BLM + Vehicle groups compared to the control and sham groups, although this difference was not statistically significant (*p* > 0.05). Similarly, while SMAD-2 levels were lower in the BLM + TS50 and BLM + TS100 groups compared to the BLM + Vehicle group, these difference were also not statistically significant (*p* > 0.05).

COL1 mRNA levels were significantly upregulated in the BLM (3.15-fold) and BLM + Vehicle (2.42-fold) groups compared to the control group (*p* < 0.0001 and *p* = 0.0001, respectively). While a downregulation in COL1 levels was observed in the BLM + TS50 groups compared to the BLM + Vehicle, this difference was not statistically significant (*p* > 0.05). Moreover, COL1 mRNA level in the BLM + TS100 group was significantly decreased compared to the BLM + Vehicle group (0.59-fold, *p* = 0.007).

Furthermore, α-SMA mRNA level was significantly upregulated in the BLM (2.23-fold) and BLM + Vehicle (2.2-fold) groups compared to the control group (*p* = 0.0001 and *p* = 0.0004, respectively). In contrast, α-SMA level was significantly downregulated in the BLM + TS50 (0.52-fold) and BLM + TS100 (0.49-fold) groups (*p* = 0.0035 and *p* = 0.001, respectively) compared to the BLM + Vehicle group.

### 2.3. Determination of TGF-β1, SMAD2, COL1, and α-SMA Protein Levels

**Figure 5:**
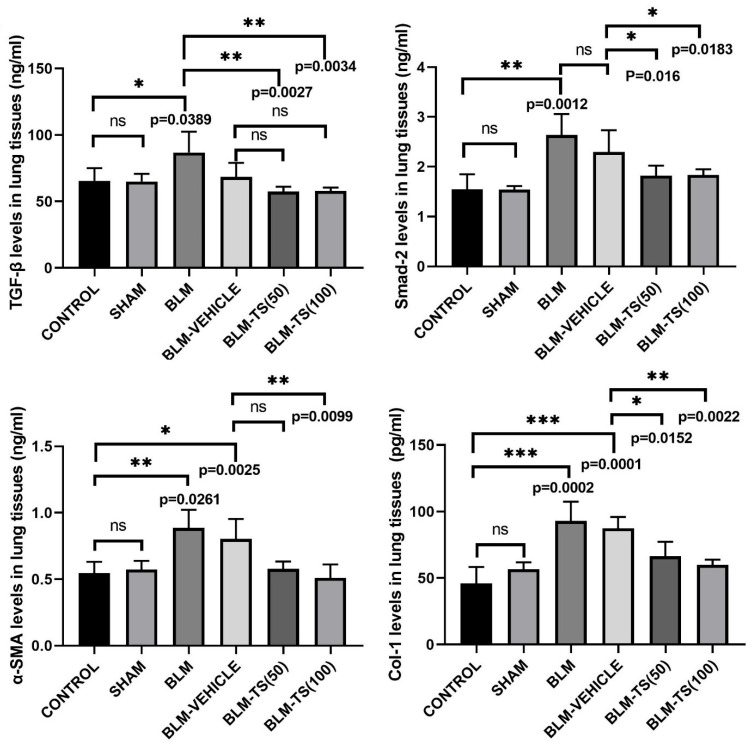
TGF-β1, SMAD2, COL1, and α-SMA protein levels. BLM administration resulted in a significant increase in TGF-β1, SMAD2, COL1, and α-SMA protein levels in lung tissue compared to the control and sham groups. While these increases were maintained in the BLM + Vehicle group, TS treatment (50 and 100 mg/kg) produced a marked reduction, particularly in fibrosis-related markers. ns: no statistically significant difference (*p* > 0.05).

The total protein amounts in the supernatants obtained from lung tissues were determined using the BCA assay, and protein levels were normalized prior to use in the experiments. Subsequently, TGF-β, SMAD2, α-SMA, and COL1 protein levels in the lung tissues were compared across the experimental groups (Figure 5). It was determined that there were no significant changes in TGF-β1, SMAD2, α-SMA, and COL1 levels when the control and sham groups were compared. In the BLM group, TGF-β protein expression levels in lung tissues were significantly increased compared to control group (*p* = 0.0389). Similarly, SMAD2, α-SMA, and COL1 protein levels were also significantly increased in the BLM group (*p* = 0.0012, *p* = 0.0261, and *p* = 0.0002, respectively), indicating that bleomycin induces a robust fibrotic response.

In the groups treated with TS (BLM + TS50 and BLM + TS100), a marked increase was observed in the levels of all fibrotic markers compared to the BLM and BLM + Vehicle groups. Specifically, TGF-β1 levels decreased significantly with TS treatment (*p* = 0.0027 and *p* = 0.0034), and a similar reduction was detected in Smad-2 levels (*p* = 0.016 and *p* = 0.0183). Furthermore, α-SMA and COL1 levels were significantly decreased following TS administration (*p* = 0.0099 for α-SMA; *p* = 0.0152 and *p* = 0.0022 for COL1), bringing values closer to those of the control/sham groups.

### 2.4. Determination of TNF-α Levels in BALF

**Figure 6:**
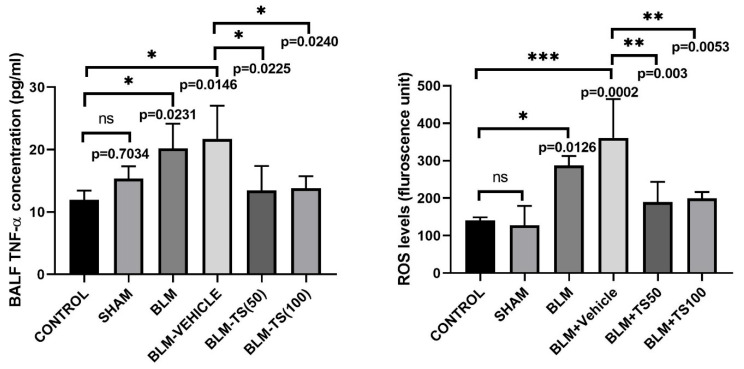
TNF-α levels in BALF and ROS levels in lung tissues. BALF TNF-α and ROS levels were significantly elevated in the BLM and BLM + Vehicle groups compared to the control and sham groups. TS treatment at both 50 and 100 mg/kg concentrations significantly decreased BALF TNF-α levels compared to the BLM and BLM + Vehicle groups. ns: no statistically significant difference (*p* > 0.05).

Figure 6 illustrates the TNF-α levels in BALFs of the control, sham, BLM, and TS groups. No statistically significant difference in TNF-α levels was observed between the control and sham groups (*p* = 0.7034). In contrast, in the group administered BLM, BALF TNF-α levels were significantly increased compared to both control and sham groups (*p* = 0.0231 and *p* = 0.0146, respectively). TNF-α levels in the BLM + Vehicle group remained comparably high relative to the BLM group, indicating that vehicle administration did not further influence BLM-induced inflammation. Conversely, in the BLM + TS50 and BLM + TS100 groups, TNF-α levels of BALFs were significantly decreased compared to both the BLM and BLM + Vehicle groups (*p* = 0.0225 and *p* = 0.0240, respectively).

### 2.5. Determination of ROS (Reactive Oxygen Species) Levels in Lung Tissue

ROS levels in lung tissue homogenates were determined fluorometrically using the DCFH-DA method, and the results are expressed in fluorescence units (Figure 6). As shown in Figure 6, ROS levels were significantly increased in the BLM and BLM + Vehicle groups compared to the control group (*p* = 0.0126 and *p* = 0.0002, respectively). Compared to the BLM + Vehicle group, ROS levels were significantly decreased in both the BLM + TS50 and BLM + TS100 groups (*p* = 0.003 and *p* = 0.0053, respectively). However, no significant difference was observed between the BLM + TS50 and BLM + TS100 groups (*p* = 0.9998).

### 2.6. Determination of Malondialdehyde (MDA) Levels in Lung Tissues

**Figure 7:**
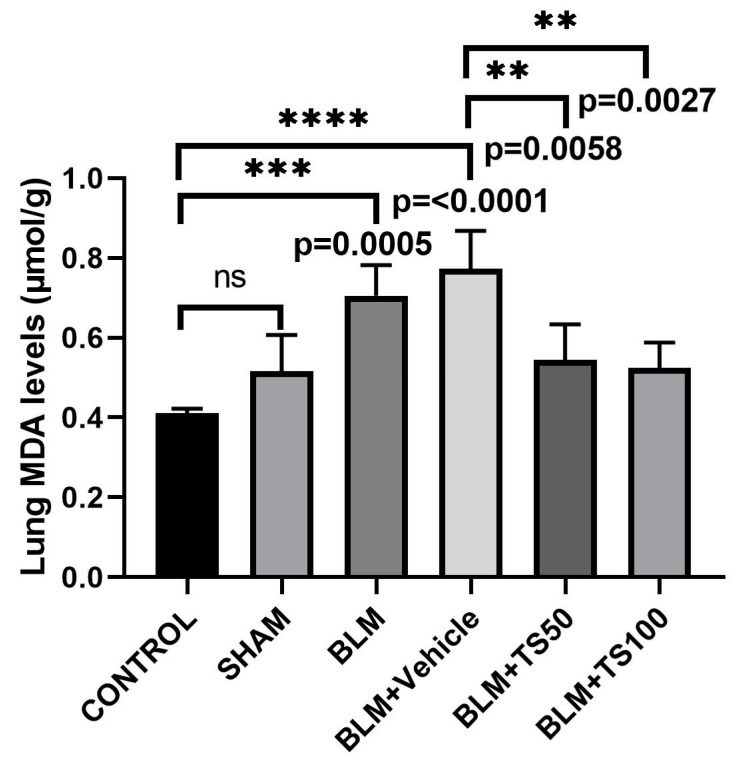
Effects of TS treatment on MDA levels in lung tissues of BLM-induced pulmonary fibrosis model. ns: no statistically significant difference (*p* > 0.05).

MDA levels in lung tissue homogenates were determined spectrophotometrically, and the results are presented in Figure 7. As shown in the Figure 7, MDA levels significantly increased in both the BLM (0.7050 µmol/g) and BLM + Vehicle (0.7728 µmol/g) groups compared to the control (0.4113 µmol/g) and sham (0.516 µmol/g) groups (*p* = 0.0005 and *p* < 0.0001, respectively). Conversely, treatment with BLM + TS50 (0.5435 µmol/g) and BLM + TS100 (0.5243 µmol/g) significantly decreased MDA levels when compared to the BLM + Vehicle group (*p* = 0.0058 and *p* = 0.0027, respectively).

### 2.7. Evaluation of DPPH Radical Scavenging Activity of TS

**Figure 8:**
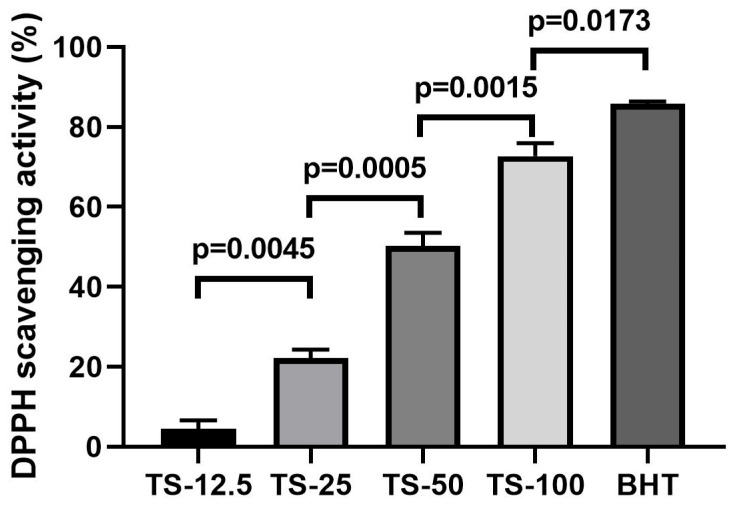
Determination of antioxidant capacity of *T. syriacus* via DPPH radical scavenging assay.

The antioxidant activity of TS was determined using the DPPH radical scavenging assay, and the results are illustrated in Figure 8. TS demonstrated potent antioxidant activity, which increased in a dose-dependent manner across the concentrations of 12.5, 25, 50, and 100 mg/mL. Specifically, the tested doses reduced the DPPH radical levels by 4.42%, 22.17%, 50.27%, and 72.72%, respectively.

### 2.8. Histopathological Evaluation and Ashcroft Scoring Results

**Figure 9:**
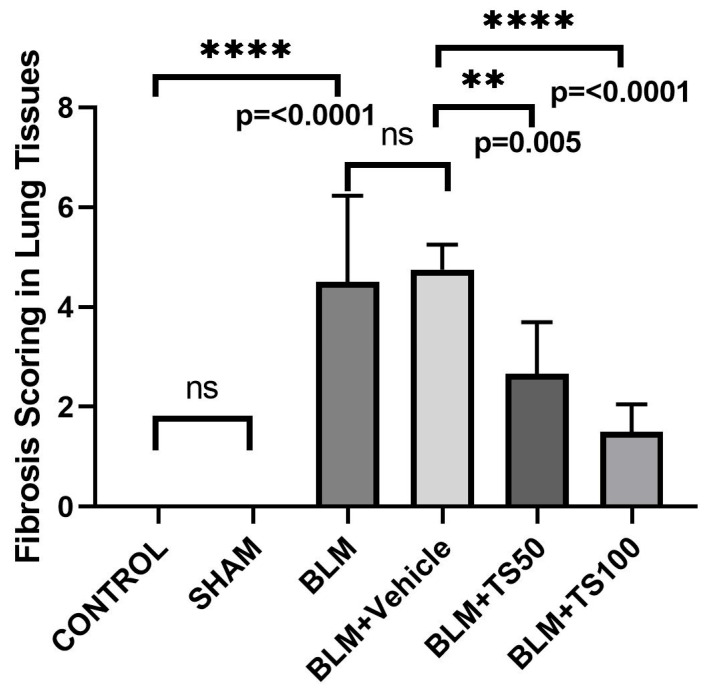
Histological fibrosis scores in lung tissues. While fibrosis scores were significantly increased in the BLM and BLM + Vehicle groups, TS treatment markedly reduced these scores. ns: no statistically significant difference (*p* > 0.05).

Lung tissue samples were histopathologically examined using Hematoxylin–Eosin (H&E) and Masson’s trichrome staining methods, and the severity of fibrosis was quantified using the Ashcroft scoring system (Table 1) [20]. The fibrosis scoring results are presented in Figure 9. Compared with the control group, the fibrosis score (severity of lung fibrosis) was significantly increased in the BLM and BLM + Vehicle groups (*p* < 0.0001 for both). In contrast, fibrosis scores were significantly decreased in the BLM + TS50 and BLM + TS100 groups compared to the BLM + Vehicle group (*p* = 0.005 and *p* < 0.0001, respectively).

**Figure 10:**
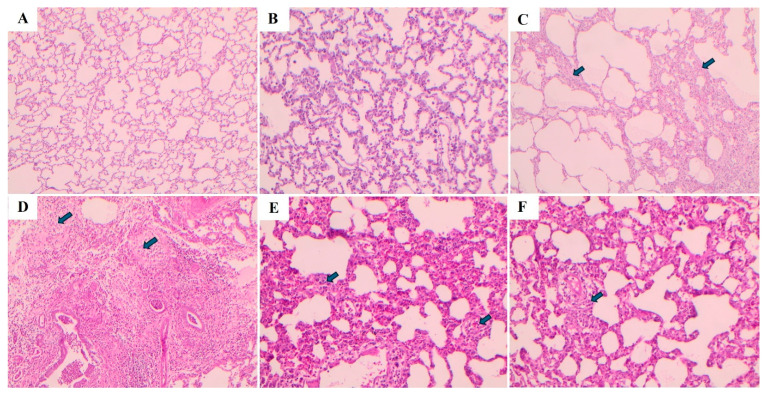
Histopathological images of lung tissues stained with hematoxylin and eosin. (**A**) control, (**B**) sham, (**C**) BLM, (**D**) BLM + Vehicle, (**E**) BLM + TS50, (**F**) BLM + TS100. The arrows indicate areas of alveolar septa thickening.

**Figure 11:**
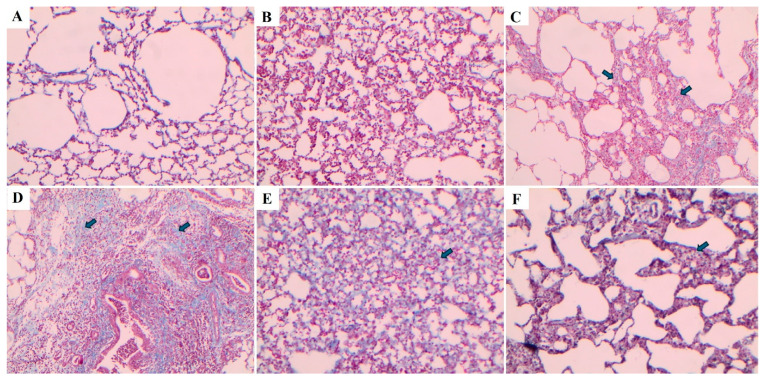
Masson’s trichrome-stained lung tissue sections. (**A**) control, (**B**) sham, (**C**) BLM, (**D**) BLM + Vehicle, (**E**) BLM + TS50, (**F**) BLM + TS100. The arrows indicate areas of alveolar septa thickening.

Lung tissues were stained using Hematoxylin–Eosin (H&E) and Masson’s trichrome staining methods, and the images are presented in Figure 10 and Figure 11, respectively. In the control and sham groups, the Ashcroft score was determined as “0”. H&E staining revealed no fibrotic burden in the alveolar septa, and no septal thickening, cellular inflammation, or fibrotic changes were observed. The normal alveolar architecture of the lung parenchyma was fully preserved. Similarly, Masson’s trichrome staining showed no increase in connective tissue, indicating that the lung tissue maintained a completely normal histological structure.

In the BLM group, the Ashcroft score was evaluated as “4”. H&E staining revealed variable fibrotic changes between areas in the alveolar septa. Limited, small foci of fibrotic masses were observed in less than 10% of the microscopic fields of the lung tissue. Masson’s trichrome staining showed a marked increase in connective tissue, consistent with the presence of these limited fibrotic area. In the BLM + Vehicle group, the Ashcroft score was determined as “5”. H&E staining revealed marked thickening of the alveolar septa and dense fibrotic bands. Large, contiguous fibrotic masses were observed in 10–50% of the microscopic fields. Despite substantial damage to the parenchymal structure, the overall architecture of the lung was preserved. Masson’s trichrome staining showed a pronounced increase in connective tissue, consistent with an advanced fibrotic pattern.

In the BLM + TS50 group, the Ashcroft score was evaluated as “3”. H&E staining revealed septal thickening and contiguous fibrotic bands throughout the microscopic fields, with septal thickness reaching approximately three times that of normal. Alveoli were mildly enlarged and sparsely distributed, but no prominent fibrotic masses were observed. Masson’s trichrome staining showed a mild increase in connective tissue. In the BLM + TS100 group, the Ashcroft score was determined as “2”. H&E staining demonstrated marked thickening of the alveolar septa (approximately three-fold) with nodular, non-contiguous fibrotic areas. Although alveolar spaces were slightly enlarged and sparse, no significant fibrotic masses were detected. Masson’s trichrome staining revealed a very mild increase in connective tissue.

### 2.9. Evaluation of Serum Biochemical Parameters

**Figure 12:**
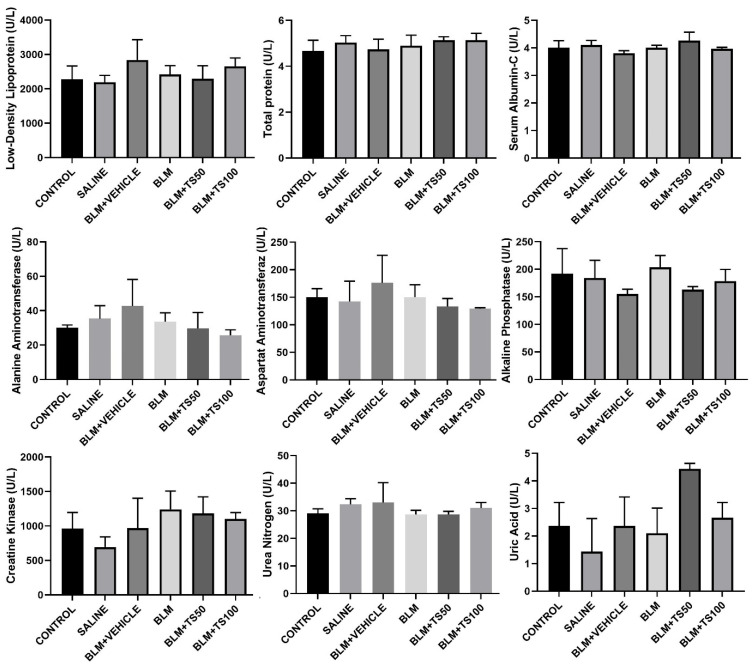
Serum biochemical parameters in experimental groups. Serum levels of low-density lipoprotein (LDL), total protein, serum albumin, alanine aminotransferase (ALT), aspartate aminotransferase (AST), alkaline phosphatase (ALP), creatine kinase (CK), urea nitrogen, and uric acid were measured to evaluate systemic toxicity and organ function including liver, kidney, heart.

Serum biochemical parameters were analyzed to assess potential systemic toxicity and organ functions in the experimental groups, and the results are presented in Figure 12. The parameters examined include low-density lipoprotein (LDL), total protein, serum albumin, alanine aminotransferase (ALT), aspartate aminotransferaz (AST), alkaline phosphatase (ALP), creatine kinase (CK), urea nitrogen, and uric acid.

LDL levels were similarly distributed among the groups, indicating that neither BLM nor TS administration significantly affected the lipid profile. No significant differences were observed in total protein and serum albumin levels among the control, sham, BLM, and BLM + TS groups, suggesting that the treatments did not adversely impact general protein metabolism and nutritional status.

Evaluation of liver function markers ALT, AST, and ALP revealed a slight increasing trend in the BLM and BLM + Vehicle groups; however, these changes were not indicative of marked hepatotoxicity. In the TS-treated groups (BLM + TS50 and BLM + TS100), ALT and AST levels tended to be lower compared to the BLM group, while ALP levels were comparable to those of the control group. No significant differences were observed in creatine kinase levels, a marker cardiac injury. Kidney function indicators, urea nitrogen and uric acid levels, remained within physiological limits in all groups. Specifically, urea nitrogen levels in the TS-treated groups were similar to those of the control group, and no clinically significant increase in uric acid levels was observed.

### 2.10. GC–MS Analysis of TS

GC–MS analysis identified a total of 16 compounds in the TS, with the identified compounds accounting for 100% of the total oil composition (Table 2). As indicated in Table 2, the essential oil is mostly composed of oxygenated monoterpenes and monoterpene hydrocarbons. The most dominant compound was thymol, constituting more than half of the total oil components at 63.5%. Thymol was followed by p-cymene (17.6%) and carvacrol (13.4%). The original chromatogram of the GC-MS analysis of TS is included in the Supplementary Material (Figure S1).

## 3. Discussion

Bleomycin is an agent used in cancer treatment; however, as a side effect, it also triggers pulmonary fibrosis and affects approximately 10% of patients taking the drug [21]. Therefore, identifying agents or sources with potential therapeutic effects on pulmonary fibrosis, whether arising idiopathically or as a consequence of drug-induced toxicity, such as from bleomycin, is of considerable importance. Therefore, this study was designed to molecularly evaluate the potential therapeutic effects of TS rich in bioactive compounds with potent antioxidant and anti-inflammatory activities against a bleomycin-induced pulmonary fibrosis model.

Medicinal plants possess a broad spectrum of biological activities, including antioxidant [22], anticancer [23], anti-inflammatory [24], antidiabetic, and antimutagenic effects [25], among others. Furthermore, plants and their isolated compounds have been reported to exhibit significant antifibrotic effects in experimental models of pulmonary fibrosis [21]. Previous studies have reported that *Thymus* species may play a role in suppressing pulmonary and liver fibrosis [18,19]. Nevertheless, information about the mechanistic role of the antifibrotic effects of *Thymus* species remains limited, and elucidating their impact on the TGF-β/SMAD pathway is essential for identifying new potential sources for combating pulmonary fibrosis. In this context, the present study molecularly investigated the antifibrotic effect of TS in a bleomycin-induced lung fibrosis model. Following the daily administration of TS at concentrations of 50 and 100 mg/mL for 28 days, animal body weights were significantly higher compared to the BLM and BLM + Vehicle groups. Conversely, a decrease in body weight was observed only in the BLM and BLM + Vehicle groups relative to the other groups. Consistent with our findings, previous studies have demonstrated that animal body weights decrease in BLM-treated groups and following the administration of antifibrotic agents [26]. Furthermore, this study determined that lung index scores increased in the BLM and BLM + Vehicle groups, whereas they decreased in the TS-treated groups. The increase in lung index scores in BLM groups and their subsequent reduction in treatment groups are consistent with previously reported data [26].

Among the key markers of fibrosis, cytokines such as TNF-α and Interleukin-1β play a role [27]. TNF-α binds to TNFR1 receptors, activating signaling pathways that lead to the production of reactive oxygen species (ROS). ROS, such as hydrogen peroxide (H_2_O_2_), superoxide anion (O_2_•^−^), hydroxyl radical (HO•), and nitric oxide (NO), cause oxidative stress, resulting in tissue damage. On the other hand, the interaction between oxidative stress and TGF-β plays a critical role in inducing fibrosis. By increasing ROS production, TGF-β triggers oxidative stress [3]. In the present study, it has been demonstrated that TNF-α, a marker of bleomycin-induced lung inflammation, was increased in the bronchoalveolar lavage fluid (BALF) collected from the lungs of the BLM and BLM + Vehicle groups. Furthermore, the increase in TNF-α levels was significantly suppressed in a concentration-dependent manner following treatment with TS. Additionally, ROS levels were elevated in the BLM and BLM + Vehicle group tissues, whereas they were reduced in the BLM + TS50 and BLM + TS100 groups. These findings suggest that TS administration significantly suppressed the TNF-α-related inflammatory response, indicating that TS may possess anti-inflammatory effects in bleomycin-induced lung inflammation. Studies conducted with various species of the *Lamiaceae* have consistently reported that TNF-α levels are suppressed in pulmonary fibrosis models [28]. To the best of our knowledge, no previous studies have demonstrated the effects of *Thymus* species on TNF-α and ROS levels in lung tissues or BALF. In parallel with the ROS results, MDA (malondialdehyde) levels were found to be elevated in the lung tissues of the BLM groups, whereas they were significantly reduced in the TS-treated groups. Lipid peroxidation generates free radicals that function as toxic and reactive aldehyde metabolites, with an increase in malondialdehyde (MDA) levels serving as a key indicator of this process. MDA levels are modulated by endogenous antioxidant mechanisms [29]. Furthermore, animal toxicity studies have demonstrated that the administration of *Thymus* species significantly attenuates MDA levels [30]. Consequently, it can be concluded that TS, through its potent antioxidant properties, effectively mitigates bleomycin-induced oxidative stress and the associated tissue damage.

The antioxidant capacity of TS was initially evaluated using the DPPH radical scavenging assay, a widely accepted method for determining the free radical scavenging potential of natural compounds. The present study demonstrated that TS exhibits a concentration-dependent scavenging activity against DPPH radicals. This potent antioxidant activity is primarily attributed to the high content of monoterpenes, such as thymol, p-cymene and carvacrol, which are well-documented for their ability to donate hydrogen atoms to free radicals, thereby neutralizing them [31,32].

Various plants belonging to *Lamiaceae* have been shown to suppress the organ fibrosis by inhibiting the increase in TGF-β-induced SMAD2 and α-SMA levels [28]. Additionally, Chen et al. [32] reported that fibrosis is suppressed by targeting the TGF-β/Smad pathway with phytochemicals such as phenolics, terpenoids, ketones, and aldehydes, thereby reducing the levels of pathway-associated α-SMA and COL1. Moreover, the present study elucidated that TS downregulated the mRNA and protein levels of TGF-β, SMAD2, α-SMA and COL1. The reduction in TGF-β/ SMAD2, α-SMA, and COL1 levels suggests that TS significantly attenuates the fibrotic response in the lung tissue.

The present study demonstrates that bleomycin administration induced significant fibrotic alteration in lung tissue, while TS treatment effectively mitigates the severity of fibrosis, as evidenced by Ashcroft scores and histopathological findings. Detailed histopathological examination revealed fibrotic alterations including the thickening of alveolar septa, the formation of adjacent fibrotic bands, and aberrant alveolar expansion in the BLM and BLM + Vehicle groups. Notably, these alterations were markedly attenuated following TS treatment. These findings support the potent role of TS in suppressing bleomycin-induced pulmonary fibrosis. Moreover, biochemical analysis of serum samples was performed to assess potential renal, hepatic, and cardiac toxicities. For the parameters evaluated, including low-density lipoprotein (LDL), total protein, serum albumin, alanine aminotransferase (ALT), aspartate aminotransferase (AST), alkaline phosphatase (ALP), creatine kinase (CK), urea nitrogen, and uric acid, no significant alterations were observed between the control, BLM, and TS treatment groups. Collectively, these findings indicate that neither bleomycin nor TS treatment induces systemic biochemical toxicity. Consequently, it was considered that TS treatment exhibits a favorable safety profile regarding hepatic, renal, and metabolic functions in this model, suggesting that its therapeutic effects are independent of systemic adverse effects.

Finally, the chemical composition of TS was elucidated using GC–MS analysis. The primary constituents were identified as thymol (63.5%), p-cymene (17.6%), and carvacrol (13.4%), with thymol accounting for more than half of the total oil content. Previous studies have identified *Thymus* species components as thymol and carvacrol; however, it has been determined that this order has changed. Tümen and Baser [33] reported the main compounds of *Thymus* species as thymol (49.04%), carvacrol (15.87%), and p-cymene (6.75%). On the other hand, various studies have shown that TS is richer in carvacrol [34]. Hence, it is considered that the composition of TS can vary depending on factors such as collection region, collection time, analysis method, and extraction type. Previous studies have reported that thymol possesses strong antioxidant and anti-inflammatory activities [35]. Hussein et al. [36] report that thymol modulates miR-29a/TGF-β expression, suppressing oxidative stress and inflammation, thereby inhibiting bleomycin-induced pulmonary fibrosis. Additionally, the same study reported that it exhibited anti-inflammatory effects by suppressing TNF-α and antioxidant activity by decreasing MDA levels. p-Cymene is the monoterpene with the highest quantity after thymol. It has been reported that this compound plays a protective role in lung damage induced by lipopolysaccharides by suppressing inflammatory cytokines [37]. Furthermore, p-cymene suppresses the formation of liver fibrosis by reducing inflammatory markers (NF-κB, IL-1β, IL-6, TGF-β1) [38]. Interestingly, Yamada et al. [39] detected the levels of five volatile compounds in the exhaled breath of patients with idiopathic pulmonary fibrosis (IPF) and healthy controls. Compared to the control group, four (acetoin, isoprene, ethylbenzene, and an unknown compound) of the peaks detected in IPF patients were identified, and their levels were found to be increased. The other peak was attributed to p-cymene, and it was observed that the amount of p-cymene had decreased. Based on this, it is hypothesized that p-cymene, which has antioxidant and anti-inflammatory activity, may play a protective role against IPF in the human body [39]. Carvacrol has been reported to exhibit antioxidant properties by suppressing malondialdehyde (MDA) production and increasing glutathione levels, as well as anti-inflammatory effects through modulation of inflammatory markers such as TNF-α and IL-6 [40]. Moreover, it has been demonstrated that carvacrol plays a role in attenuating renal and hepatic fibrosis through inhibition of the TGF-β/SMAD signaling pathway [41,42]. Finally, a reduction in inflammation and fibrotic lesions has been shown in a bleomycin-induced pulmonary fibrosis model following carvacrol treatment [43]. Taken together, these findings suggest that the antifibrotic effects observed in our study may be largely attributed to the high carvacrol content of *T. syriacus* essential oil.

## 4. Materials and Methods

### 4.1. Collection, Drying, and Essential Oil Extraction of T. syriacus

*T. syriacus* was collected on 30 May 2024, from the Gaziantep-Nizip region, Turkey. The species was taxonomically identified by Associate Professor Dr. Mustafa Pehlivan from the Department of Biology, Gaziantep University, and was assigned an individual herbarium number (MPH2024-1). The aerial parts of the collected plants were washed and then air-dried in a dark, well-ventilated area. For essential oil extraction, the dried plant material was ground into a fine powder and subjected to hydrodistillation using a Clevenger apparatus for 3 h, yielding 2.09% (*w*/*w*) essential oil. The obtained oil was stored at +4 °C until further use in experiments.

### 4.2. Pulmonary Fibrosis Model

This study was approved by the Adıyaman University Experimental Animals Ethics Committee (protocol number: 2024/044). Male Wistar albino rats, aged 8–12 weeks and bred at the Adıyaman University Experimental Animals Center, were used in the experiments. The animals were maintained under a controlled 12 h light/dark cycle and provided with ad libitum access to drinking water and standard rat chow.

**Figure 13:**
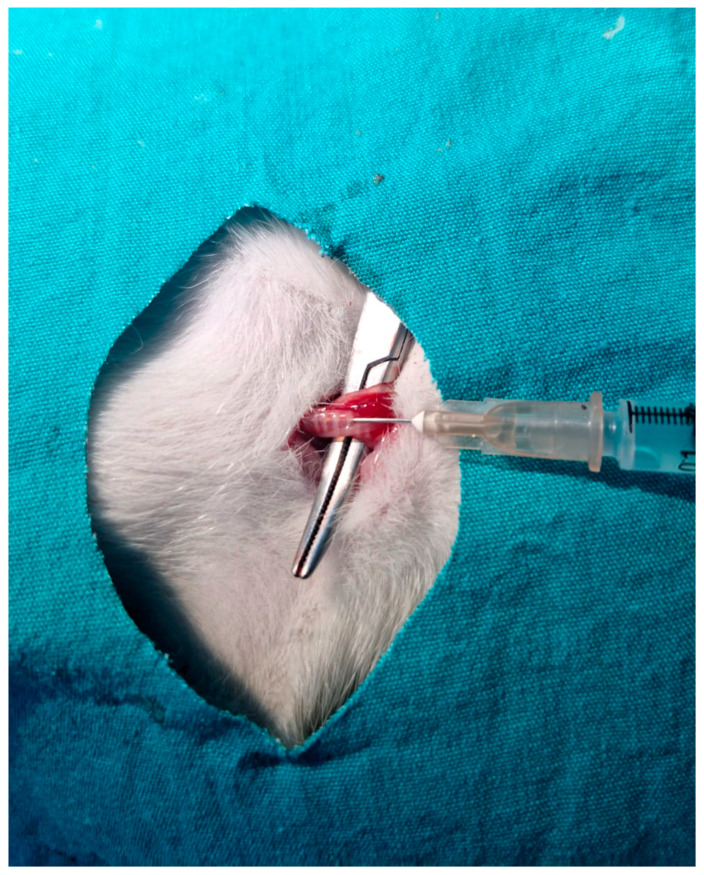
Establishment of the pulmonary fibrosis model by intratracheal administration of bleomycin.

Pulmonary fibrosis was induced in rats via intratracheal administration of bleomycin. Briefly, the pulmonary fibrosis model was established by administering bleomycin at a dose of 5 mg/kg, dissolved in 100 µL of sterile saline, directly into the trachea [26]. Rats were anesthetized, and a small midline incision was made at the cervical region to access the trachea, after which the solution was delivered using an insulin syringe (Figure 13).

**Figure 14:**
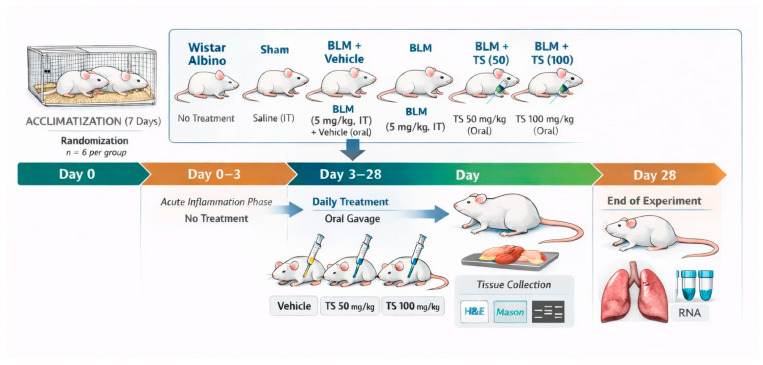
Experimental design and timeline for investigating the effects of TS in a bleomycin-induced lung fibrosis model.

Animal groups:

The concentrations of TS utilized in the present study were selected based on concentration ranges reported in previous literature [19,44,45]. The experimental workflow is illustrated in Figure 14.

Group 1: This group serves as the untreated control, in which no interventions were performed.

Group 2: The sham group consisted of rats that underwent surgical exposure of the trachea only and received 0.1 mL of saline (the bleomycin vehicle).

Group 3: Bleomycin group: pulmonary fibrosis was induced by surgically exposing the trachea and administering bleomycin at a dose of 5 mg/kg.

Group 4: Bleomycin + Vehicle group: rats that received bleomycin were additionally administered 0.5 mL of sunflower oil, which served as the vehicle for TS.

Group 5: Bleomycin + TS group (50 mg/kg/day): TS was administered via gavage starting 3 days after bleomycin administration.

Group 6: Bleomycin + TS group (100 mg/kg/day): TS was administered via gavage starting 3 days after bleomycin administration.

On day 28 of the experiment, all animals were sacrificed under ketamine and xylazine anesthesia, and lung tissues were isolated. The thorax was opened via a midline incision under anesthesia, and the trachea was cannulated using a plastic catheter attached to a 10 mL syringe. Bronchoalveolar lavage fluid (BALF) was collected by instilling 5 mL of sterile saline into the lungs five times, with gentle massage to facilitate fluid recovery. The BALF was then centrifuged at 300× *g* for 10 min at 4 °C to obtain the supernatant for biochemical analyses.

For histopathological examinations, one-third of the lung tissue was fixed in 10% formalin. The remaining tissue samples were stored at −80 °C until further molecular analyses. In addition, blood samples were collected for biochemical analyses and stored at +4 °C. 

### 4.3. Determination of Animal Body Weights

The body weight of the animals was assessed at the beginning and the end of the experiment. The percentage of body weight gain for each group was calculated using the following equation:

BW (%) = [(fBW − iBW)/fBW] × 100

where BW is the percentage of body weight gain, fBW is the final body weight at the end of the experimental period, and iBW is the initial body weight at the beginning of the experimental period.

### 4.4. Determination of the Lung Index

After the sacrifice of the rats, the lungs, including the trachea, were completely excised and weighed at the end of the experiment to determine the lung-to-body weight ratio. The lung index was calculated using the following formula:

Lung Index = Lung Weight/Body Weight

### 4.5. Determination of Total Protein Levels

Lung tissue samples weighing 100 mg each were homogenized in 1/10 (*w*/*v*) cold saline using a homogenizer. The homogenate was then centrifuged at 10,000 g for 10 min, and the supernatant was collected. Total protein levels in the supernatant were determined using a BCA-ELISA kit (KTD3010-EN, Abbkine, Wuhan, China). The total protein concentrations in the lung samples were measured according to the manufacturer’s protocol.

### 4.6. Evaluation of the Pro-Inflammatory Cytokine TNF-α in BALF

The levels of the pro-inflammatory cytokine tumor necrosis factor alpha (TNF-α) in the BALF were quantified using a commercial rat-specific enzyme-linked immunosorbent assay (ELISA) kit (Cat no: ER1393, FineTest, Wuhan, China), according to the manufacturer’s instructions.

### 4.7. Determination of TGF-β1, SMAD2, Col1, and α-SMA Protein Levels

The levels of transforming growth factor beta (TGF-β, cat no:ER1378, FineTest, China), mothers against decapentaplegic homolog 2 (SMAD2, cat no: ER06645, FineTest, China), collagen type I (COL1, ER0848, FineTest, China), and alpha-smooth muscle actin (α-SMA ER1457, FineTest, China) in lung tissue homogenates were quantified using commercial rat-specific enzyme-linked immunosorbent assay (ELISA) kits, following the respective manufacturer’s protocols.

### 4.8. RNA Isolation from Lung Tissues and Preparation of cDNA Samples

For RNA isolation from lung tissues, QuickEX Tissue Total RNA Extraction Kit (Cat. No. NGE024–50 NucleoGene, Istanbul, Türkiye) was used. The lung tissues were placed into sterile tubes and homogenized using TissueLyser LT–Bead Mill Tissue Homogenizer (QIAGEN Sample and Assay Technologies, Düsseldorf, Germany). RNA was isolated from the tissues, and the extracted RNA was subsequently reverse transcribed into cDNA using FIREScript^®^ RT cDNA synthesis kit (Cat. No. 06-15-00050, Solis BioDyne, Tartu, Estonia) reacted at 25 °C for 10 min, 50 °C for 5 min, 85 °C for 5 min, following the manufacturer’s instructions. cDNA synthesis reactions were performed at MiniAmp™ Thermal Cycler (Applied Biosystems™, Waltham, MA, USA). The forward and reverse primer sequences for TGF-β, SMAD2, COL1, and α-SMA are listed in Table 3.

### 4.9. Analysis of Gene Expression Using Quantitative Real-Time PCR (qRT-PCR)

Gene expression analyses were performed using with QIAGEN Rotor-Gene Q Real-Time PCR system (QIAGEN Sample and Assay Technologies, Germany). HOT FIREPol^®^ EvaGreen^®^ qPCR Mix Plus (Cat. No. 08-24-00001, Solis BioDyne, Estonia) reacted at 95 °C for 12 min, 40 cycles 95 °C for 15 s, 60 °C for 20 s, 72 °C for 20 s, according to the manufacturer’s protocol.

### 4.10. Assessment of MDA Levels of Lung Tissues

MDA levels in lung tissues were determined according to the method described by Heath and Packer [44,46], with minor modifications. Briefly, 0.1 g of lung tissue was homogenized in 5 mL of cold PBS, and the homogenate was centrifuged at 10,000 *g* for 10 min. Subsequently, 1 mL of the supernatant was mixed with 4 mL of a solution containing 20% TCA and 0.5% thiobarbituric acid. The mixture was incubated in a water bath at 95 °C for 30 min, immediately cooled in an ice bath, and centrifuged again at 10,000 *g* for 5 min. Absorbance was then measured at 532 and 600 nm. MDA content was calculated using an extinction coefficient of 155 mM^−1^ cm^−1^ and expressed as µmol/g tissue.

MDA (µmol/g) = [(A_532_ − A_600_)/155] × 1000

### 4.11. Determination of DPPH Scavenging Activity of TS

The free radical scavenging activity of TS was evaluated using the DPPH assay as described by Saint-Cricq de Gaulejac et al. [47], with minor modifications. Briefly, 0.1 mL of 12.5, 25, 50 and 100 mg/mL concentrations of TS was added to 2.9 mL of a DPPH solution. The reaction mixture was incubated in the dark at room temperature for 45 min. Following incubation, 300 µL of the mixture was transferred into a 96-well microplate, and absorbance was measured at 517 nm using a microplate reader (Biochrom, Cambridge, UK). BHT (20 µg/mL) was used as positive control [48].

DPPH scavenging activity (%) = [(A_blank_ − A_sample_)/A_blank_] × 100

### 4.12. Assessment of Histopathological of Lung Tissues

Lung specimens were fixed in 10% neutral-buffered formalin for 7 days. Following fixation, the specimens were dehydrated through a graded alcohol series, cleared in xylene, and embedded in paraffin. Sections were cut at 7 μm using a manual microtome (RM 2125; Leica Instruments, Nussloch, Germany). Sections were mounted on slides, deparaffinized, rehydrated, and then stained using a Masson’s trichrome kit (Bio-Optica, Milan, Italy). For histopathological evaluation, sections were photographed at 40× magnification using a light microscope (Axiocam ERc5s; Carl Zeiss, Göttingen, Germany) equipped with a digital camera.

### 4.13. Biochemical Analyses of Blood Samples

For biochemical analyses, blood samples collected in EDTA-containing tubes were centrifuged at 4000 rpm for 10 min at 4 °C to separate the serum. The serum was then aliquoted and stored at −20 °C until analysis. Clinical biochemistry parameters were measured using an automated analyzer (Siemens, Erlangen, Germany). The liver profile included total protein, alkaline phosphatase (ALP), alanine aminotransferase (ALT), and aspartate aminotransferase (AST); the renal profile included urea and uric acid; and the cardiac profile included creatinine kinase (CK).

### 4.14. Determination of the Phytochemical Content of TS

The volatile constituents of TS were identified using gas chromatography–mass spectrometry (GC–MS) (Agilent, Santa Clara, CA, USA), while their relative percentages were determined by gas chromatography with a flame ionization detector (GC–FID). The sample was diluted in hexane to 10% (*w*/*w*), and 1 µL was injected using a 40:1 split ratio. GC–FID analyses were performed on an Agilent 7890B system equipped with an Agilent HP-Innowax capillary column (60 m × 0.25 mm i.d., 0.25 µm film thickness), with injector and detector temperatures set at 250 °C. The oven temperature was programmed from 60 °C (10 min) to 220 °C at 4 °C/min (held 10 min), then to 240 °C at 1 °C/min, for a total runtime of 80 min, with helium as the carrier gas at 0.7 mL/min. GC–MS analyses were carried out on the same system coupled with an Agilent 5977B Mass Selective Detector under identical chromatographic conditions; the injector was set at 250 °C, the ion source at 230 °C, using electron ionization at 70 eV and scanning m/z 35–450. Volatile compounds were identified by comparison of mass spectra with Wiley 9 and NIST 11 libraries, and their relative percentages were calculated from GC–FID peak areas using area normalization without correction factors. Relative retention indices (RRIs) were calculated against a series of n-alkanes (C7–C40) on a polar column.

### 4.15. Statistical Analyses

Statistical analyses were performed to assess differences among multiple experimental groups. One-way analysis of variance (ANOVA) was used to evaluate overall significance, followed by Tukey’s post hoc test for pairwise comparisons to determine specific group differences. All analyses were conducted using GraphPad Prism version 8.0.2 (GraphPad Software, San Diego, CA, USA). Statistical significance was denoted as *, **, ***, and ****, corresponding to *p* < 0.05, *p* < 0.01, *p* < 0.001, and *p* < 0.0001, respectively. Graphical representations of the data were generated using the same software to facilitate visualization and interpretation of group differences.

## 5. Conclusions

In this study, the antifibrotic properties of TS on the TGF-β1/SMAD2 signaling pathway and its associated fibrotic markers, COL1 and α-SMA, were investigated in a bleomycin-induced pulmonary fibrosis model using molecular, biochemical, and histopathological approaches. The results demonstrated that TS suppresses the TGF-β1/SMAD2 pathway, targets COL1 and α-SMA expression, and consequently inhibits the mechanisms underlying fibrotic formation in lung tissue. In addition, TS administration was shown to exert no toxic effects on vital organs such as the heart, liver, and kidneys.

To the best of our knowledge, no previous studies have reported the effects of TS on pulmonary fibrotic signaling pathways; therefore, the present study is the first report in this field and provides evidence that TS may serve as a potential therapeutic agent by exerting anti-inflammatory and antifibrotic effects in bleomycin-induced pulmonary fibrosis, thereby contributing to and guiding future research in this area.

Nevertheless, this study has some of limitations. These include the lack of a positive control treatment such as pirfenidone. Another limitation of the study is the lack of measurement of both enzymatic and non-enzymatic antioxidant levels, including superoxide dismutase, catalase, and glutathione.

Moreover, while the present study focused solely on the TGF-β/SMAD signaling pathway, it is suggested that other fibrosis-related mechanisms, particularly the Wnt/β-catenin signaling pathway, should also be investigated following TS treatment and compared with the TGF-β/SMAD pathway to provide a more comprehensive understanding of its antifibrotic mechanisms

## Figures and Tables

**Table 1:** Ashcroft scale for histological grading of lung damage.

Grade	Histological Findings
0	Normal lung,
1	Alveoli partly enlarged and rarefied, but no fibrotic masses present
2	Alveoli partly enlarged and rarefied, but no fibrotic masses
3	Alveoli partly enlarged and rarefied, but no fibrotic masses
4	Single fibrotic masses (fibrotic area covering ≤ 10% of the microscopic field)
5	Confluent fibrotic masses (fibrotic area covering > 10% and ≤50% of the microscopic field).
6	Large contiguous fibrotic masses (fibrotic area covering > 50% of the microscopic field)
7	Alveoli nearly obliterated with fibrous masses but a few air bubbles remain
8	Microscopic field completely occupied by fibrotic masses

**Table 2:** Compounds identified in the GC–MS analysis of TS.

No	RRI	Compound	%
1	1025	α-Pinene	0.5
2	1028	α-Thujene	0.3
3	1063	Camphene	0.2
4	1174	α-Terpinene	0.8
5	1195	Limonene	0.2
6	1204	β-Phellandrene	0.1
7	1207	1,8-Cineole	0.1
8	1270	p-Cymene	17.6
9	1449	1-Octen-3-ol	0.2
10	1605	Carvacrol methyl ether	0.7
11	1609	Terpinen-4-ol	1.5
12	1705	Borneol	0.5
13	1731	β-Bisabolene	0.2
14	1850	p-Cymen-8-ol	0.2
15	2166	Thymol	63.5
16	2199	Carvacrol	13.4
		Total	100
		Monoterpene hydrocarbons	19.7
		Oxygenated Monoterpenes	79.9
		Other	0.4

RRI: Relative retention index. Only compounds with a relative abundance ≥ 0.1% are reported.

**Table 3:** Primer sequences for target mRNA analysis in lung tissue.

Gapdh	Forward	CACAGTCAAGGCTGAGAATG
	Reverse	GCATTGCTGACAATCTTGAG
Tgf-β	Forward	TACGCCAAAGAAGTCACCCG
	Reverse	GTGAGCACTGAAGCGAAAGC
SMAD2	Forward	AAGGAACAAAAGGTCCGGGG
	Reverse	AGACCCACCGGCTGATTTTT
COL1	Forward	GCAAGAGGCGAGAGAGGTTT
	Reverse	ACCAACGTTACCAATGGGGC
α-SMA	Forward	TTCGTGACTACTGCTGAGCG
	Reverse	CTGTCAGCAATGCCTGGGTA

## Data Availability

The original contributions presented in this study are included in the article. Further inquiries can be directed to the corresponding author.

## Supplementary Materials

The following supporting information can be downloaded at https://www.mdpi.com/article/10.3390/ijms27031401/s1.

## Abbreviations

The following abbreviations are used in this manuscript:

TS	Thymus syriacus essential oil
TNF-α	Tumor Necrosis Factor alpha
TGF-β	Transforming Growth Factor beta
Col-1	Collagen type I
α-SMA	Alpha-Smooth Muscle Actin
Smad	SMAD family signaling proteins
GC–MS	Gas Chromatography–Mass Spectrometry
EDTA	Ethylenediaminetetraacetic Acid
DPPH	2,2-diphenyl-1-picrylhydrazyl
PCR	Polymerase Chain Reaction
BALF	Bronchoalveolar Lavage Fluid
BCA	Bicinchoninic Acid
MMP	Matrix Metalloproteinase
MDA	Malondialdehyde
