# Supplementary figures and images for: Antifibrotic Effects of Thymus syriacus Essential Oil in Bleomycin-Induced Pulmonary Fibrosis via Suppression of the TGF-β1/Smad2 Axis
Source: Int J Mol Sci. 2026 Jan 30;27(3):1401. doi: 10.3390/ijms27031401 (PMC12898269; doi:10.3390/ijms27031401)

Figure S1. GC-MS Chromatogram of TS (GC-20260013)

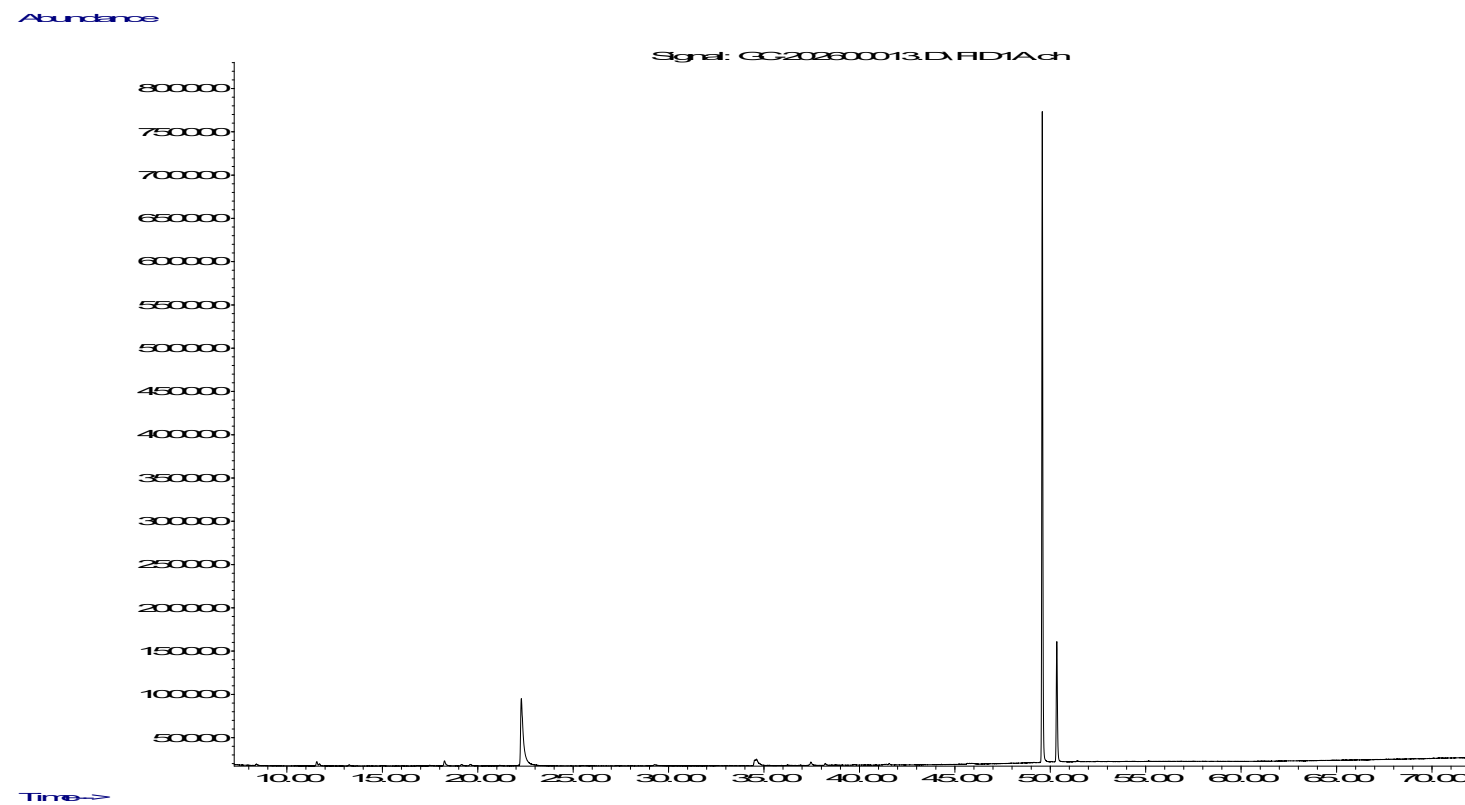

Supplement: Supplementary file 1 [file ijms-27-01401-s001.zip › ijms-4110195-supplementary.pdf]
